# A Novel Human Mutation in the SLC9A1 Gene Results in Abolition of Na^+^/H^+^ Exchanger Activity

**DOI:** 10.1371/journal.pone.0119453

**Published:** 2015-03-11

**Authors:** Xiuju Li, Larry Fliegel

**Affiliations:** Department of Biochemistry, University of Alberta, Edmonton, Alberta, Canada; University of Saskatchewan, CANADA

## Abstract

The SLC9A1 gene, the Na^+^/H^+^ exchanger isoform 1 is the principal plasma membrane Na^+^/H^+^ exchanger of mammalian cells and functions by exchanging one intracellular proton for one extracellular sodium. The human protein is 815 amino acids in length. Five hundred N-terminal amino acids make up the transport domain of the protein and are believed to form 12 transmembrane segments. Recently, a genetic mutation of the Na^+^/H^+^ exchanger isoform 1, N266H, was discovered in a human patient through exome sequencing. We examined the effect of this mutation on expression, targeting and activity of the Na^+^/H^+^ exchanger. Mutant N266H protein was expressed in AP-1 cells, which lack their endogenous Na^+^/H^+^ exchanger protein. Targeting of the mutant protein to the cell surface was normal and expression levels were only slightly reduced relative to the wild type protein. However, the N266H mutant protein had no detectable Na^+^/H^+^ exchanger activity. A histidine residue at this location may disrupt the cation binding site or the pore of the Na^+^/H^+^ exchanger protein.

## Introduction

The Na^+^/H^+^ exchanger is a plasma membrane pH regulatory protein present in all human mammalian cells. While there are several isoforms of the protein, the NHE1 (Na^+^/H^+^ exchanger type one) isoform is ubiquitous and critical in cell growth, proliferation, differentiation, and apoptosis. NHE1 is an integral membrane protein. It has an N-terminal membrane domain of approximately 500 amino acids that transports ions, and a cytoplasmic C-terminal domain of 315 amino acids that regulate activity. NHE1 functions to maintain pH homeostasis in normal and neoplastic cells. It promotes ion transport by extruding one intracellular proton in exchange for one extracellular sodium ion. The inwardly directed Na^+^ gradient drives transport [1,2].

Human NHE1 is a total of 815 amino acids long. The complete structure of human NHE1 is not known however the protein has been the subject of several studies (reviewed in [1]). Early studies using cysteine scanning accessibility predicted a topology of 12 transmembrane segments [3]. NHE1 is distantly related to the bacterial Na^+^/H^+^ exchanger NhaA and the crystal structure of NhaA has been solved [4].

A role for NHE1 has been implicated in cardiovascular disease [5] and in the progression of several forms of cancer [6]. Additionally, previous studies in animals have shown that deletion of this protein results in a severe phenotype. Mice with a mutation in NHE1 causing lack of functional expression exhibit ataxia and have recurrent seizures by 2 to 3 weeks of age, which is usually accompanied by early death [7,8]. Deletion of NHE1 from mice also results in neuronal damage in the cerebellum and brainstem [8]. We [9] have recently characterized a single base pair inherited mutation in Gly^305^ of the NHE1 gene (SLC9A1) that results in an Arg substitution. The presence of this mutation causes a near complete mistargeting, and loss of proton pumping by NHE1. In a consanguineous family, when homozygous, this mutation results in an inherited phenotype of ataxia and deafness accompanied by developmental disorders. Thus it is clear that mutations in NHE1 can result in severe developmental disorders.

With the advent of next generation sequencing platforms, the cost of genome and in particular exome sequencing, has been greatly reduced. Most alleles that underlie Mendelian inherited disorders change protein coding sequences and though a few are well known, many rare disorders are uncharacterized [10]. For the SLC9A1 gene there has been little study in this area. In this contribution, we examine the effect of a novel mutation in the NHE1 transmembrane domain. The results are the first description of this mutation and the resultant effects on protein activity, expression and targeting.

## Materials and Methods

### Materials

2', 7-bis (2-carboxyethyl)-5(6) carboxyfluorescein acetoxymethyl ester (BCECF-AM) was from Molecular Probes, Inc. (Eugene, OR). Sulfo-NHS-SS-biotin was purchased from Pierce and synthetic DNA was purchased from IDT (Coraiveille, IA, USA). Streptavidin agarose was obtained from Thermo Scientific. Anti HA (hemagglutinin) tag primary antibody was from Pierce. GAM-HRP was obtained from Jackson ImmunoReseach Lab Inc(Bio/Can, Mississauga, Canada). Other chemicals that were used were of analytical grade and were purchased from Fisher Scientific (Ottawa, ON, Canada), Sigma (St. Louis, MO, USA) or BDH (Toronto, ON, Canada). The plasmid pYN4+ contains the human NHE1 protein with a HA tag and has been described earlier [11].

### Site specific mutagenesis

Whole exome sequencing revealed the presence of a de novo mutation N266H found in SLC9A1. This was present in a patient that presented with spastic diplegia, seizures, autism, intellectual disability, behavioral problems and possible Worster-Drought syndrome [12]. While it was not confirmed that the mutation was causal, we examined the effect of the N266H mutation to determine if this resulted in aberrant function of the NHE1 protein. Mutagenesis of NHE1 was using the plasmid pYN4+ described above [13] as described earlier [14]. DNA sequencing confirmed the fidelity of DNA amplification and the presence of the mutation.

### Cell culture and stable transfection

To characterize activity of the Na^+^/H^+^ exchanger we used AP-1 cells which are a mutant cell line derived from Chinese hamster ovarian cells that are devoid of their own NHE1 protein [15]. Stable and transiently transfected cells were made using Lipofectamine 2000 Reagent (Invitrogen Life Technologies, Carlsbad, CA, USA) essentially as described earlier [14]. The NHE1 expression plasmid, pYN4+, carries a neomycin resistance gene. This allows selection of stably transfected cells using geneticin (G418). Stable cell lines for experiments were regularly re-established from frozen stocks at passage numbers between 5–11. Results are typical of at least two stable cell lines.

### Cell surface expression

Targeting of the NHE1 protein to the cell surface was measured as described earlier [13]. Briefly, the cell surface was labeled with sulfo-NHS-SS-biotin and after solubilization, the cell surface Na^+^/H^+^ exchanger was removed with immobilized streptavidin resin. Equal amounts of unbound and total protein were examined by SDS-PAGE followed by western blotting measuring immunoreactive (HA-tagged) NHE1 protein. The image density on western blots was estimated using Image J 1.35 software (National Institutes of Health, Bethesda, MD, USA). It was not possible to efficiently and reproducibly elute proteins bound to immobilized streptavidin resin so we examined total and unbound protein rather than protein bound to immobilized streptavidin. The amounts of the NHE1 protein on the plasma membrane were estimated by comparing both HA-immunoreactive species (upper and lower bands).

### SDS-PAGE and immunoblotting

The expression levels of NHE1 were confirmed by immunoblotting samples of cells with antibodies against the HA tag present on the C-terminus of NHE1. Samples were run on 10% SDS-PAGE gels and were electrotransferred to nitrocellulose membranes. The primary antibody was anti-HA monoclonal antibody; the secondary antibody used for signal detection was peroxidase-conjugated goat anti-mouse antibody. Reactive protein was detected on X-ray film using the Amersham enhanced chemiluminescence western blotting and detection system.

### Intracellular pH measurement

To measure the activity of the Na^+^/H^+^ exchanger 2',7'-Bis-(2-Carboxyethyl)-5-(and-6)-Carboxyfluorescein was used to quantify intracellular pH (pH*i*) recovery after an acute acid load as described earlier [14]. Cells were grown to *≈*90% confluence on coverslips and fluorescence was measured using a PTI Deltascan spectrofluorometer with parameters described earlier. Briefly, acute acidosis was induced by ammonium chloride (50 mM × 3 min) addition and withdrawal. The first 20 sec of recovery from acidification and was measured as ΔpH/sec after calibration of intracellular pH fluorescence for each sample [14]. Results shown are the mean ± S.E of at least 6 experiments and statistical significance was determined using the Wilcoxon Signed-Rank test.

## Results

**Fig 1:**
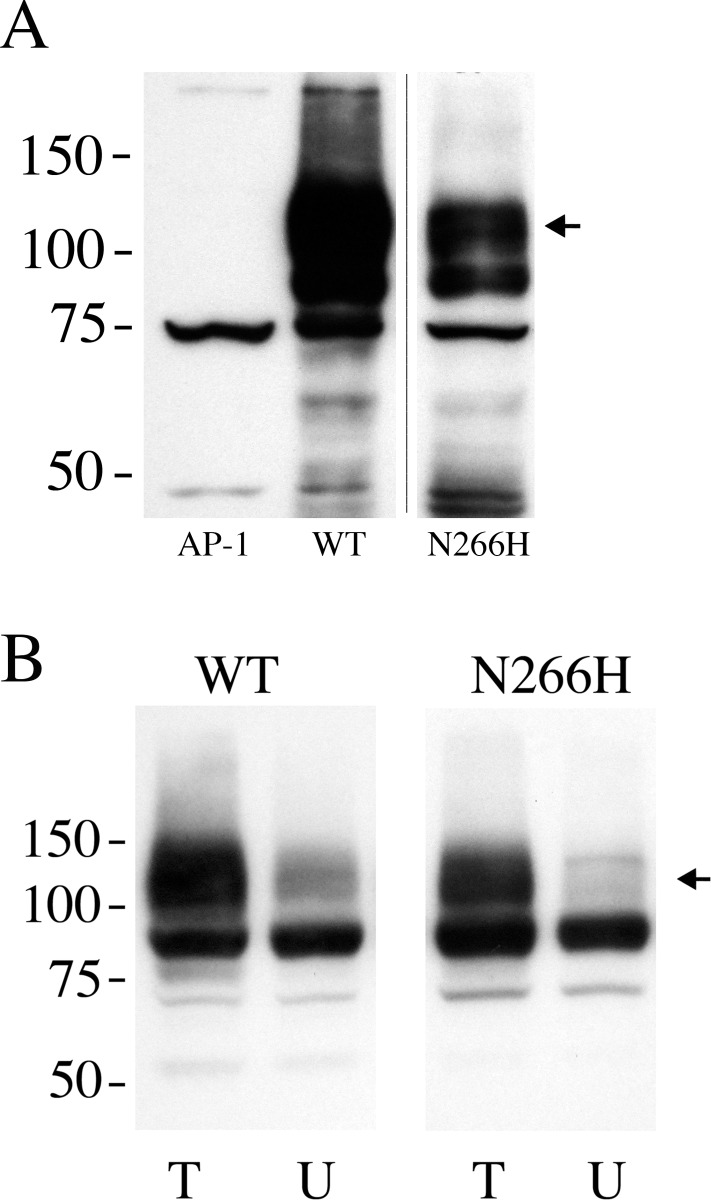
Analysis of expression and targeting of wild type (WT) NHE1 and mutant (N266H) protein. **A**, Western blot of whole cell lysates of stable cell lines expressing WT Na^+^/H^+^ exchanger or N266H mutant protein. 100 μg of total protein was loaded in each lane. The sample was immunoblotted with anti-HA tag antibody. The arrow indicates the position of full length glycosylated NHE1 protein. AP-1 is a cell lysate from mock transfected AP-1 cells. Results are typical of 5 stable cell lines. **B**, Surface localization of NHE1 in AP-1 cells expressing WT and N266H mutant. Equal amounts of total cell lysate (T) and unbound intracellular lysate (U) were examined by Western blotting with anti-HA antibody to identify NHE1 protein as described in the “Materials and Methods”. WT and N266H are cell lines stably expressing wild type NHE1 and mutant NHE1 respectively. Results are the mean ± the S.E. n = at least 3 determinations. The arrow indicates the position of full length glycosylated NHE1 protein.

To determine the putative effect of the N266H mutation on the NHE1 protein we mutated cDNA for the NHE1 protein. This was then stably expressed in AP-1 cells that lack their own NHE1 protein. The NHE1 protein contained a HA tag for detection. We have earlier determined that this tag does not affect NHE1 function [14]. Initially we examined the expression of the N266H mutant in AP-1 cells using Western blot analysis (Fig. 1A). Cells stably transfected with wild type NHE1 displayed the characteristic pattern of a 105–100 kDa immunoreactive protein and a second smaller protein of 80–90 kDa. The smaller band is the caused by reduced, or an absence of glycosylation of the NHE1 protein [14]. AP-1 cells not transfected with NHE1, showed no such immunoreactive pattern. Cells transfected with the N266H mutant DNA also showed the same characteristic pattern of protein expression. The amount of protein expressed was slightly reduced. Quantification of the amount of NHE1 protein showed that it was reduced by about 1/3 (to 63.8 +/- 3.4% of control levels).

We have earlier made mutations of the NHE1 protein to study its structure and function [14,15]. Some of these resulted in protein mistargeting, with reduced amount of NHE1 protein on the cell surface. To determine if the N266H mutation resulted in aberrant targeting of the NHE1 protein to the cell surface, we examined cell surface targeting of the protein. The results (Fig. 1B) demonstrated that there was no significant decrease in the cell surface targeting of the N266H mutant protein.

**Fig 2:**
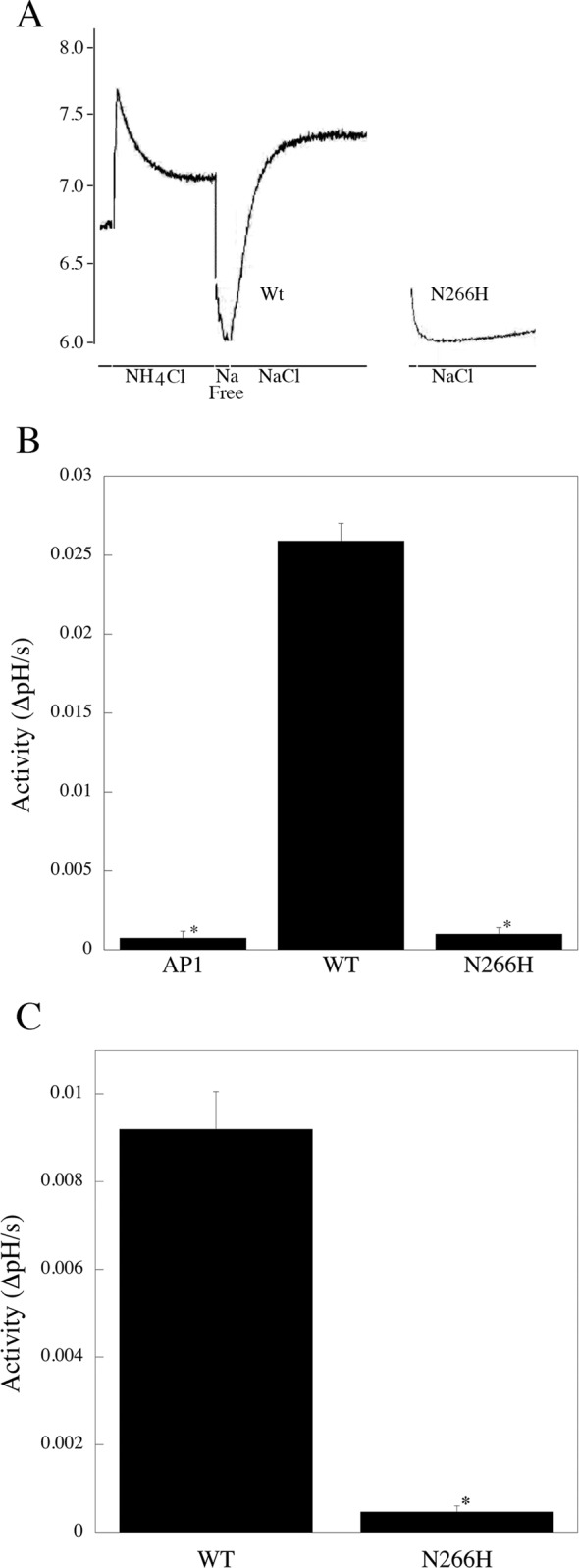
Characterization of activity of wild type (WT) NHE1 and mutant (N266H) protein. **A,** Example of Na^+^/H^+^ exchanger activity of stable cell lines containing wild type NHE1 and N266H NHE1 mutant protein. NHE1 protein activity was assayed in stably transfected AP-1 cells as described in the “Materials and Methods”. Results are typical of 5 independently made cell lines. For ease of viewing, only the recovery from acidosis is shown for the N266H protein. NH_4_Cl, treatment with ammonium chloride. After NH_4_Cl treatment there was a brief “Na Free” treatment to induce acidosis. NaCl, recovery from acidosis was in NaCl containing buffer. **B,** Summary of activity of WT and mutant ((N266H) NHE proteins in stably transfected cells. Activity was measured after ammonium chloride prepulse as described in the “Materials and Methods”. Results are change in intracellular pH/s. * indicates significantly different from wild type *P < 0.001, n>6. **C,** Summary of activity of WT and mutant (N266H) NHE proteins in transiently transfected cells. * indicates significantly different from wild type *P < 0.001, n = 8.

We then used a fluorometric assay to determine the activity of the NHE1 mutant protein relative to that of the wild type NHE1 protein. Fig. 2A illustrates examples of NHE1 activity of a stable cell line expressing either wild type, or the N266H mutant protein. Fig. 2B shows a summary of the results. The N266H protein showed no significant NHE1 activity and only a background current that was equivalent to untransfected AP-1 cells. In contrast the wild type NHE1 protein showed a typical large NHE1 activity that we have noted earlier [14,15].

To confirm the results obtained with the stable cell line expressing the N266H protein we did a similar experiment using transient transfection of AP-1 cells with either wild type or mutant (N266H) DNA. Western blotting confirmed that both wild type and N266H protein were expressed (not shown). Measurement of NHE activity (Fig. 2C) once again demonstrated that transfection of the wild type cDNA resulted in significant NHE1 activity, while transfection with the N266H mutant once again resulted in no activity.

**Fig 3:**
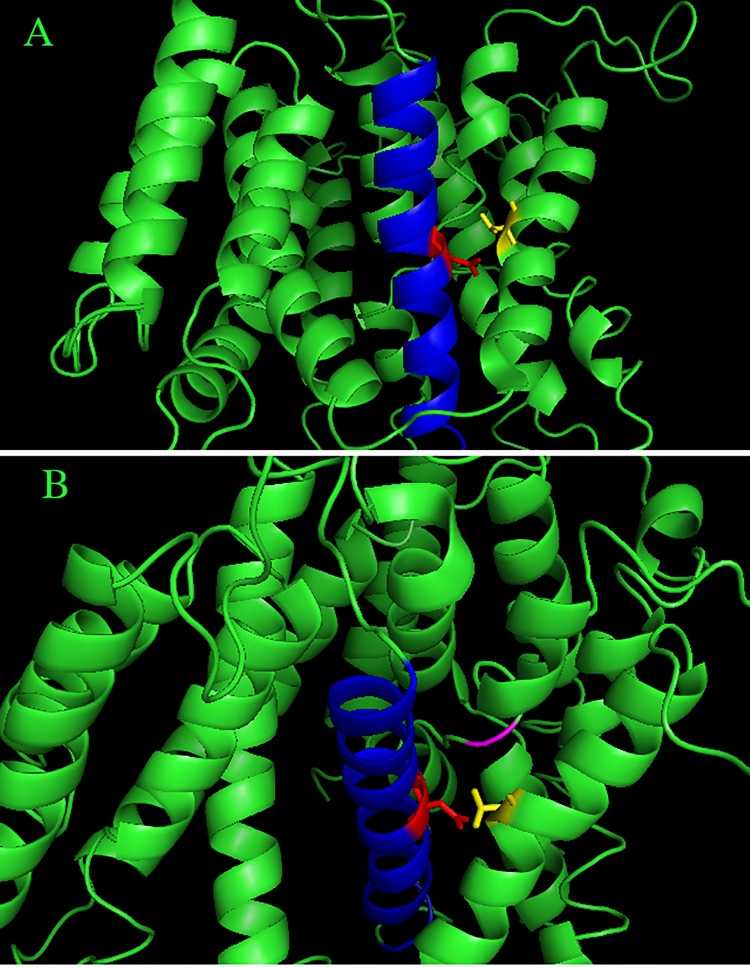
Molecular model of N266 within the NHE1 protein. Position of N266 within the molecular model of NHE1 proposed by Landau et al. [16]. TM VII of NHE1 (amino acids 254–277) are highlighted in blue, N266 and its side chain are shown in red. The side chain of amino acid T197 of TM V is shown in yellow. Ser^235^ of the extended region of TM IV is indicated by magenta color. **A,** side view through the membrane. **B,** enlarged view from side and top of the membrane.

To gather insights into the mechanism by which the mutation N266H could cause a defect in NHE1 activity we examined the structure of NHE1. While the complete structure of NHE1 has not been deduced, a model of NHE1 has been elucidated [16]. Fig. 3 illustrates this model. The amino acid N266 is present in TM VII of the protein, approximately mid level within the membrane. The side chair of N266 extends partially towards TM V (Fig. 3B) but also points towards the extended region of TM IV, which is thought to be part of the critical fold of NHE1 important in transport.

## Discussion

The Na^+^/H^+^ exchanger is a critical plasma membrane protein that is involved in heart disease, tumor progression and cell growth and differentiation [5,6]. We have recently shown that an inherited genetic mutation in NHE1 results in an inactive, or nearly inactive protein and results in the disease Lichtenstein-Knorr syndrome [9]. In this case, in homozygous individuals, the mutation of Gly^305^ to Arg causes mistargeting of the protein and reduction in the level of expression. In the present study, we examined the effects of mutation of amino acid 266 from Asn to a His residue. This mutation was recently discovered through whole exome sequencing of a cohort of humans with undiagnosed genetic diseases [12]. We therefore examined the effect of the N266H mutation on the expression, targeting and activity of the NHE1 protein to determine if this mutation could have physiological consequences on the protein. We found that there was minor effect on the level of expression and no effect on targeting of the NHE1 protein, however there was a complete absence of NHE1 activity. This was in stark contrast to the G305R mutation [9] that resulted in greatly reduced expression and almost complete mistargeting of the protein, which likely accounted for the reduced activity that was found. This suggested that the nature of these two mutations was quite different from one another. The nature of the N266H mutation is likely a direct effect on the active site of the protein, without affecting structure. In previous studies, we have found that mutations that alter the conformation of Na^+^/H^+^ exchangers tend to cause aberrant targeting of these proteins [17,18].

We have previously characterized a number of transmembrane segments of NHE1 and often found that some mutations in transmembrane segments affect expression levels and targeting of the protein. Sometimes these affects account for all of the loss of NHE1 activity, but at other times they do not [14,17,19]. In the case of the N266H mutation, effects on targeting or expression do clearly not account for the loss of activity. In some cases we have found that mutation of residues affects the level of glycosylation of NHE1 which often correlates with mistargeting of the protein [14,17,19]. This occurred with the G305R protein [9] but was not evident in the N266H mutation in the present study, in this case there was a complete absence of activity of the mutant N266H NHE1 protein and glycosylation levels were similar to that of the control.

Though there is some dispute about the topology of NHE1, (reviewed in [2]) amino acid Asn266 putatively maps to transmembrane segment VII of the NHE1 protein [3]. We have previously studied this region of the NHE1 protein [15,20]. This transmembrane segment is predominantly alpha helical with a break in helix mid membrane. As part of a study on this transmembrane segment, amino acid 266 was mutated to alanine [15]. Similar to the present study, this mutation had no or little effect on expression of the protein or on surface targeting, but also eliminated activity of the protein. Changing this amino acid to cysteine [20] also had minor effects on expression level and targeting, but eliminated NHE1 activity. Clearly this amino acid is important to NHE1 activity. We have also suggested earlier that amino acid Asn^266^ lies along the ion transduction pore of the protein. This could account for the essential role that this amino acid plays. Landau et al. [16] have also suggested that this highly conserved residue is part of, or near the cation binding site. Asp^267^ is adjacent to Asn^266^. Asp^267^ and its charge are critical to NHE1 function, and are thought to be involved in cation binding which is critical for the function of NHE1 [15,16]. Our own recent work [21] has suggested that at least part of TMVII is in close association with TM VI however Asn^266^ was oriented away from TM VI. Fig. 3 illustrates the location of Asn^266^ within a model of the entire NHE1 protein [16]. The model suggests that replacement of Asn^266^ with a relatively large residue such as a histidine could clearly disrupt cation binding by Asp^267^ and transport. This study, along with the previous results, demonstrate that this amino acid and this region of the NHE1 protein are essential to NHE1 function.

While the patient in which this mutation was found, exhibited similarities in phenotype as the patient with a G305R mutation 2014 [9], such as intellectual disability and impaired muscle movements, at present it was not possible to determine if the N266H was causally linked to the disorder. It is clear, however, that because of the total absence of activity of the mutant protein, a homozygous patient with this mutation would likely, result in a phenotype that would be similar to that observed in Lichtenstein-Knorr syndrome. How prone the SLC9A1 gene is to inherited genetic defects is uncertain at this time. However, we recently [22] characterized a genetic variant identified in the 1000 genomes sequencing project that had defective regulation of NHE1 activity. Further investigation into the prevalence of genetic mutations in the SLC9A1 gene appears to be called for, especially in the case of developmental disorders associated with ataxia and deafness that occur in Lichtenstein-Knorr syndrome.
